# A parathyroid cancer with soporous state, depression, and severe cognitive decline in acute renal failure

**DOI:** 10.1002/ccr3.7627

**Published:** 2023-06-29

**Authors:** Federica Vultaggio, Barbara Martino, Letizia Nitro, Emanuela Fuccillo, Giovanni Felisati, Loredana De Pasquale

**Affiliations:** ^1^ Otolaryngology Unit, Santi Paolo e Carlo Hospital, Department of Health Sciences Università degli Studi di Milano Milan Italy; ^2^ Department of Health Sciences Università degli Studi di Milano Milan Italy

**Keywords:** acute renal failure, parathyroid cancer, severe cognitive decline

## Abstract

**Key Clinical Message:**

Soporous state in acute renal failure represent an atypical presentation of parathyroid cancer. Complete prompt investigations and diagnosis have a fundamental role in the management of this disease

**Abstract:**

This report describes a case of parathyroid carcinoma (PC) with an uncommon first clinical presentation: soporous state, depression, and severe cognitive decline in association with acute renal failure. After discovering extremely high serum calcium and parathyroid hormone (PTH) levels, the diagnosis of primary hyperparathyroidism (pHPT) was made and a surgical en bloc resection was performed. After the surgical intervention, the histological examination revealed the presence of a malignant parathyroid disease, thus confirming our first preoperative suspicion.

## 1 INTRODUCTION

Parathyroid carcinoma (PC) is a rare endocrine cancer^1^, ^3^ and the most uncommon cause of primary hyperparathyroidism (pHPT). It usually induces elevated serum calcium and parathyroid hormone (PTH) levels and the clinical presentation is often characterized by severe symptoms of hypercalcemia. The diagnosis is not always immediate, especially if there is no evidence of a neck mass which may suggest this kind of disease.

The aim of this work is to describe the peculiar clinical presentation of a case of PC and to highlight the importance of suspecting a malignant parathyroid disease in the presence of a pHPT associated with peculiar biochemical and clinical features. The suspicion is essential to perform an adequate intervention at first surgical approach, as which affects the subsequent prognosis.

## 2 CASE REPORT

Here we present a male patient in his 80s with a negative family history of endocrine diseases. In the past, he received a diagnosis of anxiety disorder, mild cognitive impairment, essential hypertension, and hearing impairment.

He went to our Emergency Department, because of a soporous state associated with worsening of cognitive impairment. According to clinical reports, general examination did not describe the presence of a neck mass. Therefore, he was hospitalized at the Medicine Department to conduct medical care.

Hematological investigations revealed an important rise in the serum calcium level at 19.29 mg/dL (normal range for age 8.4–10.2 mg/dL), an extremely high value of PTH at 2146 pg/mL (normal range for age 8.7–7.6 pg/mL) and phosphorus level at 1.7 mg (normal range for age 2.5–4.5 mg/dL). The first suspicion was a pHPT.

**FIGURE 1:**
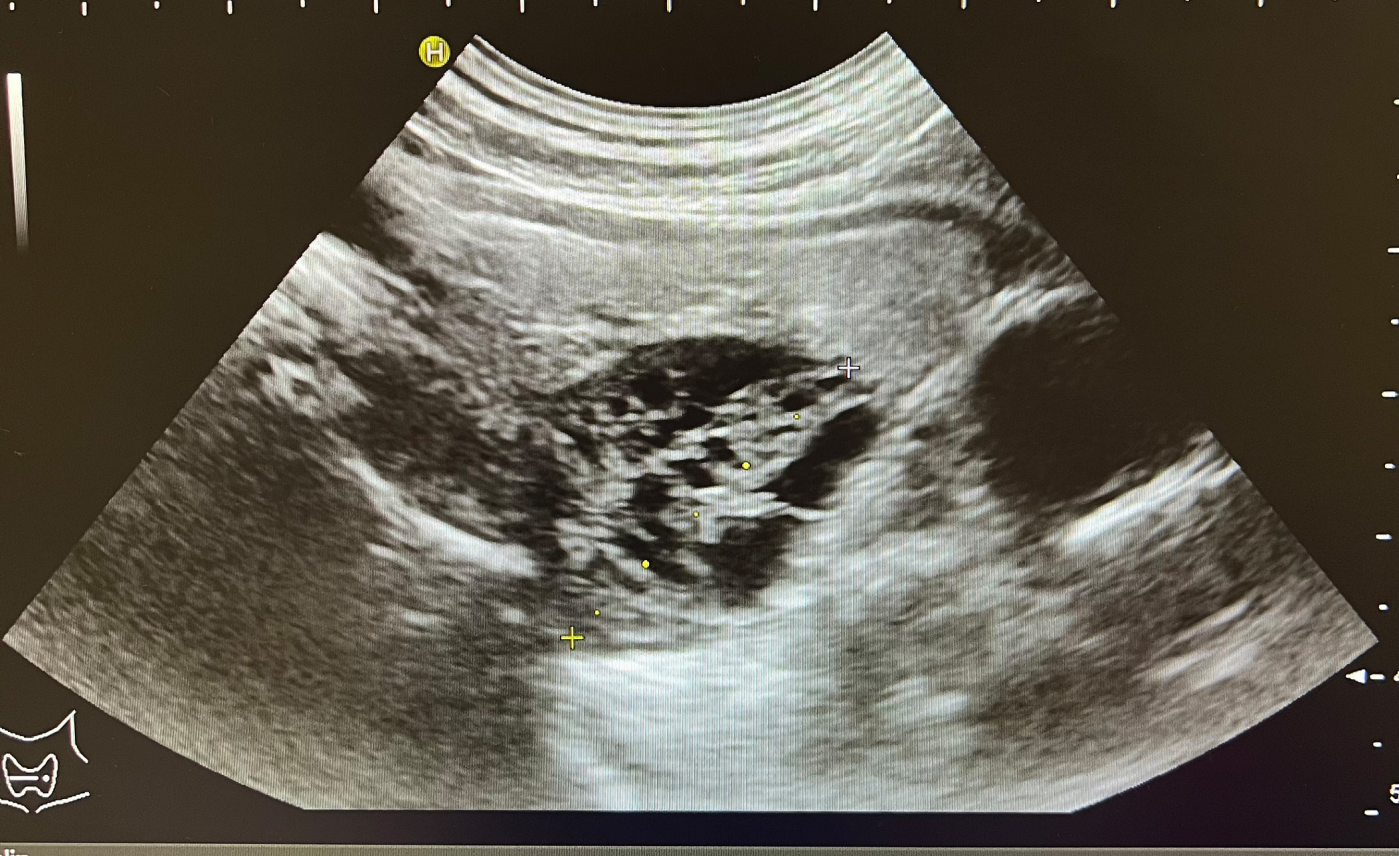
Iso‐hypo‐echoic with anechoic gaps inside rounded nodule.

During his first day of hospitalization, the urinary calcium concentration recorded was 400 mg/24 h (normal range for age <300 mg/24 h). After few days, the patient underwent a neck ultrasound that pointed out a symmetrical thyroid gland with normal size and morphology. Behind the right thyroid lobe a 28‐mm solid, iso‐hypo‐echoic with anechoic gaps inside, rounded nodule was found: it was described as indissociable from the thyroid lobe (see Figure 1).

**FIGURE 2:**
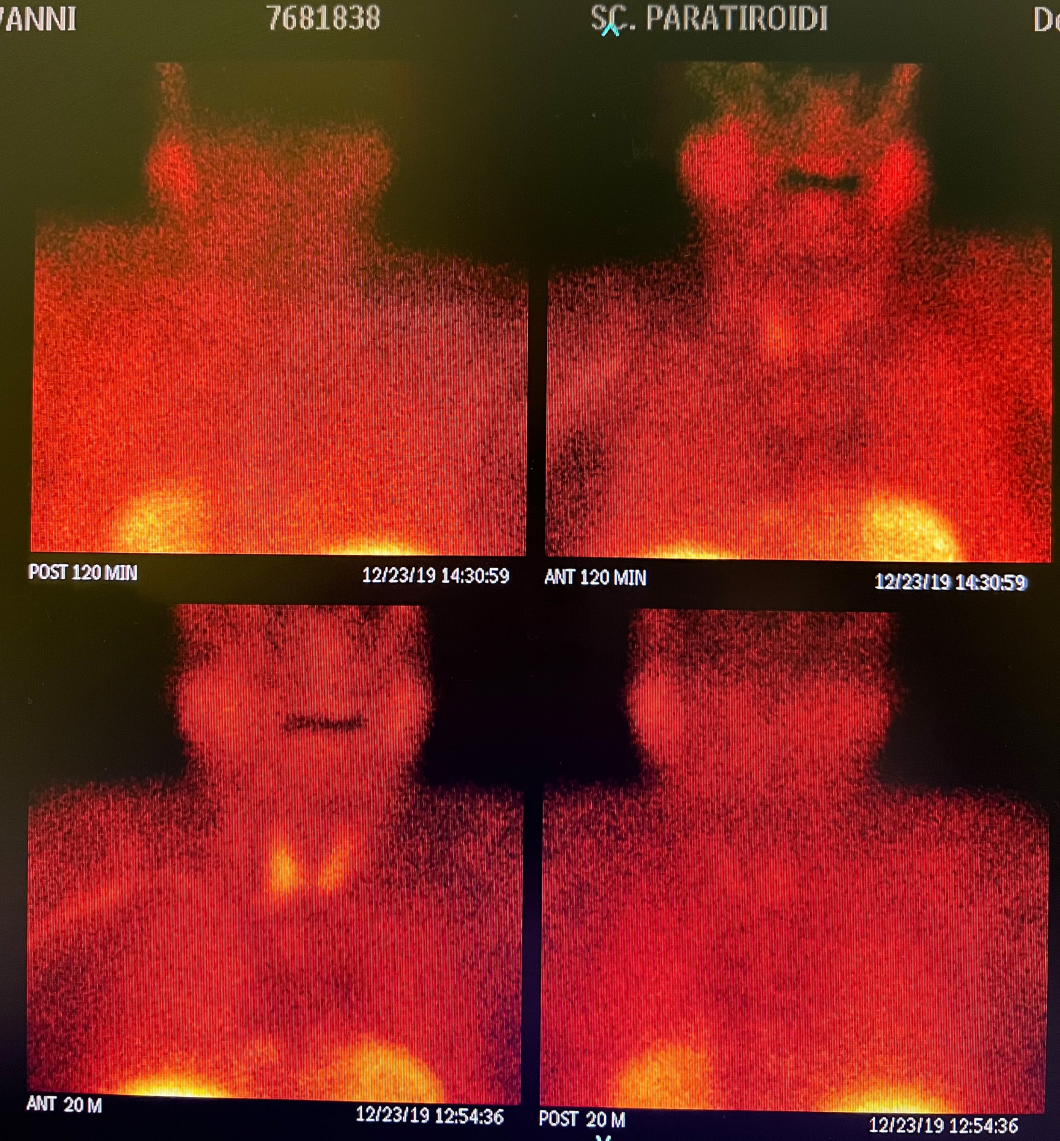
Persistent hot spot of Technetium 99 m in the lower part of the right thyroid lobe.

A parathyroid technetium‐99m sestamibi scan was performed after a week. This exam revealed a persistent hot‐spot of technetium 99m in the lower part of the right thyroid lobe and also in the posterior area, near esophagus and trachea (see Figure 2).

**FIGURE 3:**
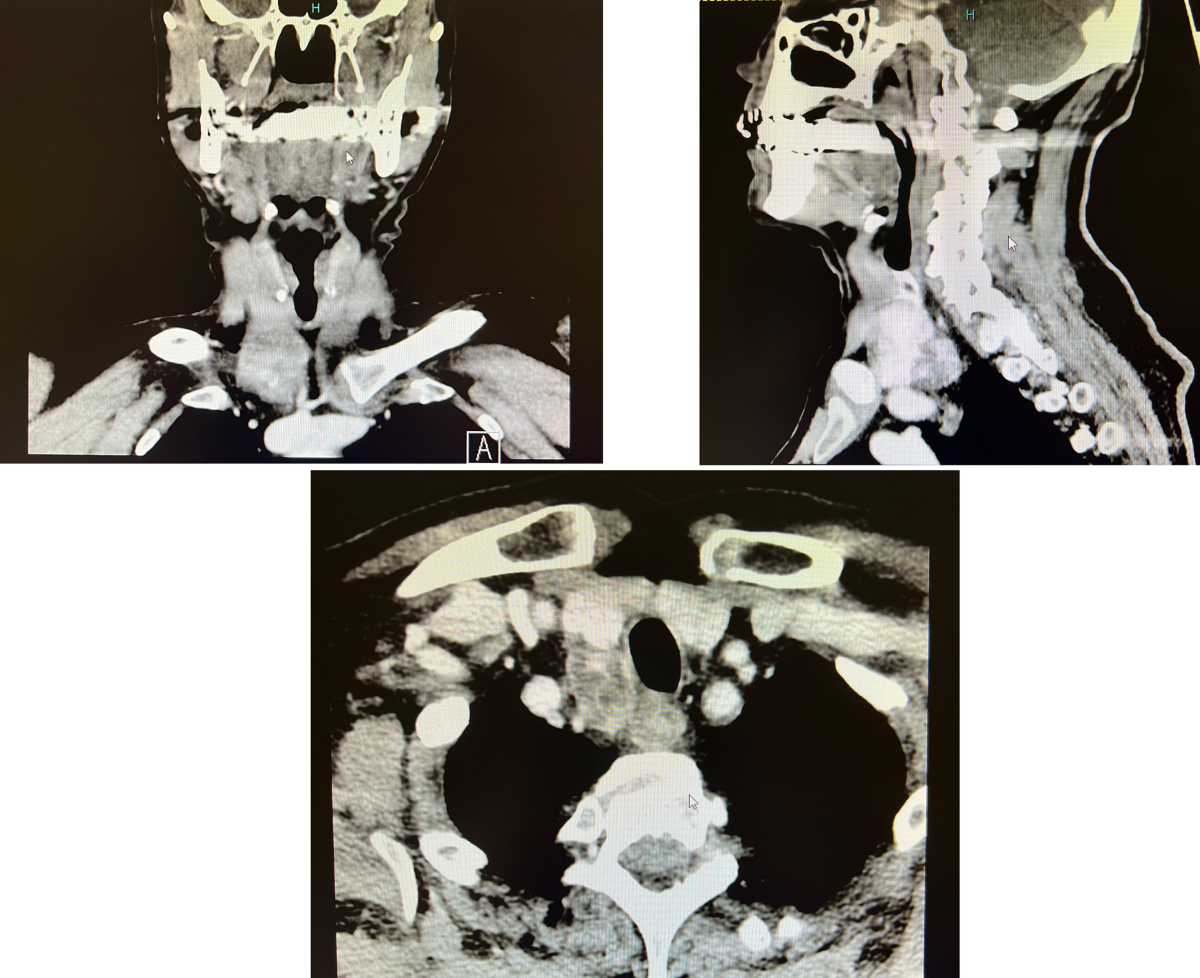
Mass in contact with right thyroid lobe, wall of esophagus and trachea without cleavage plan.

A subsequent neck CT scan executed after 1 day confirmed a mass of 31 × 21 × 31 mm characterized by irregular enhancement. This mass was in close contact with the right thyroid lobe and with the wall of the esophagus and the trachea, without evidence of a clear cleavage plane from the esophagus (see Figure 3).

To verify the esophageal infiltration, a transesophageal ultrasound‐endoscopy was performed and it pointed out an uncertain infiltration of its muscularis tunic. We could verify during the surgical treatment that the esophagus muscularis tunic was not infiltrated.

First of all, in the presence of severe hypercalcemia, it is necessary to distinguish whether primary pHPT, malignant diseases, or para‐neoplastic syndrome causes it.

In case of primary pHPT, a high PTH serum concentration will be found. Actually, the elevation of serum calcium is due to PTH‐mediated activation of osteoclasts, leading to increased bone resorption. Other causes of elevated serum calcium concentration are solid tumors, metastasis or leukemia: they may be the cause of high serum calcium level due to the activity of some cytokines and interleukin‐1. Finally, an autonomous production of parathyroid hormone‐related protein (PTHrP) can be found in the paraneoplastic syndromes: the final result is the elevation of PTH level and consequently calcemia.

With evidence of elevated serum calcium and PTH values, a diagnosis of pHPT can be made. Subsequently, clinicians should differentiate the PC‐induced pHPT and the pHPT due to benign diseases. As reported in literature, PC is a rare cause of primary hyperparathyroidism, accounting for less than 1% of cases. It should be suspected that elevated calcium and PTH serum levels, in the presence of a neck mass, or infiltration and symptoms of surrounding organs, for example dysphonia, would appear if the cancer invades recurrent laryngeal nerves.^1^, ^2^

After diagnosis of severe hypercalcemia due to pHPT, the patient started an intravenous treatment with hydration, diuretic, clodronate, calcimimetic and steroids in accordance with the nephrologist's indications. Because of the persistence of severe hypercalcemia and worsening of renal function, he was transferred to the Intensive Care Unit (ICU) for monitoring: after nephrological reevaluation, a CVC was placed to start dialysis. Subsequently, he was admitted to the Internal Medicine Unit for medical therapy. Following a new increase of serum calcium, another dialysis session was performed, and the therapy with zoledronate (4 mg iv) and cinacalcet was increased (from 60 to 120 mg/day iv). After that treatment, the calcemia level returned to the normal range with complete restoration of his cognitive functions.

Once the diagnostic process had been completed, the patient was admitted to our surgery unit where he underwent a surgical treatment about 2 weeks after the onset of symptoms: en bloc resection of the pathologic inferior right parathyroid with the right thyroid lobe and the superior macroscopically normal parathyroid. During the surgery we found a cleavage plan between esophagus and the mass, so there was no‐infiltration of muscolaris tunic, unlike the ultrasound‐endoscopy first impression. Neuromonitoring was used during the entire procedure. The ipsilateral cervical lymphadenectomy was not executed. The intraoperative values of PTH went from 749.5 pg/mL pre‐incision to 67.6 pg/mL 10 min after the removal of the pathologic gland.

Apart from the surgery no medical therapies were performed. Routine hematological investigations in the first post‐surgery day revealed a serum calcium level of 8.1 mg/dL and a serum PTH value of 9.1 pg/mL. After removing the pathologic gland, a transient condition of hypoparathyroidism was observed, but the serum level of calcium and PTH returned to the normal range after 3 months of oral calcium therapy.

**FIGURE 4:**
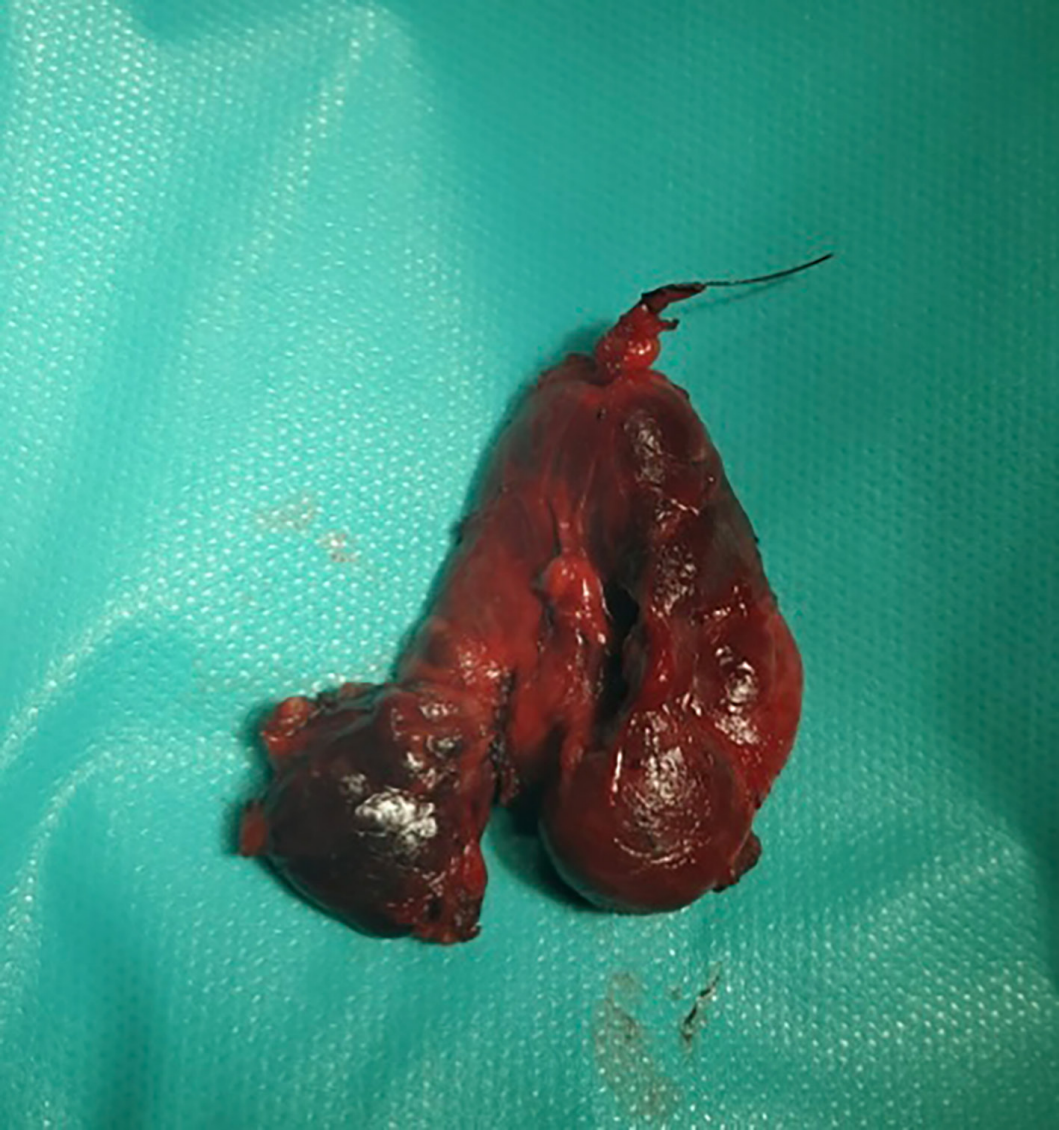
En bloc resection of pathologic inferior right parathyroid with right thyroid lobe.

Macroscopically, the pathological parathyroid gland had a diameter of 3.2 cm (see Figure 4). The weight was not defined because of the en bloc resection with the right thyroid lobe. Histological examination revealed the presence of a PC.

The follow‐up was executed at 3, 6, 9, 12, and 24 months with hematological examination; at 6, 12, and 24 months a neck ultrasound was also made. At 2‐year check‐up, the patient was disease‐free: the neck ultrasound showed no signs of local recurrence, and serum calcium and PTH values were within the normal ranges, respectively 10.2 mg/dL and 21 pg/mL. Nowadays, the patient is still alive and in good condition and regained all his cognitive functions.

## 3 DISCUSSION

The PC is an extremely rare endocrine malignancy.^3^ Its incidence is less than 1%–2% of pHPT patients, which is usually caused by a parathyroid adenoma and sometimes by primary parathyroid hyperplasia. From 1988 to 2003, 224 patients with parathyroid tumor were identified by the Surveillance, Epidemiology and End Results (SEER) cancer registry data: the incidence of PC increased from 3.58 to 5.73 per 10 million population.^4^

The etiology of the tumor remains unclear, but molecular analysis studies recognize several genes that play a central role in the molecular pathogenesis of PC, such as HRPT2 gene,^5^, ^6^ germ‐line missense variants of the parathyroid transcription factor gene GCM2^7^, ^8^ and the somatic alteration of PI3K/AKT/MTOR pathway.^9^, ^10^ Cyclin D1 amplification^11^ is also involved in the pathogenesis.

There is no difference between males and females, and the mean age of presentation is 44–54 years.

Clinical presentations are investigated in different small studies: the most common sign (65%–75%) is the elevation of serum calcium concentration above 14 mg/dL. Other signs are elevation of serum PTH concentrations, neck mass (34%–52%), bone (34%–73%) and renal disease (32%–70%), pancreatitis (0%–15%), no symptoms (2%–7%), and symptoms of local and adjacent structure invasion in case of a not functioning PC (rare form). Completely asymptomatic PC has also been described.^1^, ^2^, ^12^, ^13^

Surgical excision is the cardinal treatment. The use of intraoperative PTH (IOPTH) testing is debated: according to Medas et al^14^ it plays an important role, especially in patients with pHPT and normal PTH level. Instead, Sartori et al^15^ strongly suggest reconsidering the role of IOPTH monitoring during parathyroidectomy in patients with concordant preoperative ultrasonography and nuclear scanning.

Only after the surgical resection is it possible to have a definitive diagnosis by the analysis of the microscopic anatomy of the excised specimen.

Although this pathology is rare, it's important to suspect the diagnosis of PC pre‐operatively to undertake the best treatment for patients.

The case described in this report had an unusual clinical presentation, making the diagnosis challenging. First of all, the lack of a neck mass made the suspicion of PC less immediate. Depression and cognitive impairment are symptoms that certainly do not immediately raise suspicion of PC. Literature does not include these two among the most common symptoms of PC. Thanks to the biochemical examinations, it was possible to think about a parathyroid gland disease, which was confirmed by the neck ultrasound, the CT scan and the parathyroid–thyroid scintigraphy. Definitive diagnosis of malignancy was made after the surgical treatment and after microscopical examination.

The gold standard procedure is the en bloc resection, which means removing the parathyroid cancer with the surrounding soft tissue, the ipsilateral thyroid lobe and the adjacent structure involved by the carcinoma. This type of surgery seems to be associated with a lower rate of recurrence and death. Actually, in patients treated with complete tumor resection during the initial surgical procedure, survival rates improve to 90% and 67% at 5 and 6 years, respectively.^16^ In our case, there was no evidence of disease relapse during the follow‐up performed at 3, 6, 9, 12, and 24 months. An ipsilateral cervical lymphadenectomy can also be performed, but there is no unique opinion about that: some authors recommend it,^17^, ^18^ but not all agree with this.^19^

The patient's excellent prognosis may also be due to his classification as a low‐risk tumor, according to the Risk Schulte System,^20^ which is based on histopathologic criteria and was proposed by Talat and Shulte in 2010. It represents an important tool to predict the survival and the recurrence of the PC. The system classifies pathologies as low or high risk. Low risk tumors invade the capsule and adjacent soft tissues, while high risk ones involve vascular structures and vital organs.

The good prognosis of our patient has a limit: the follow‐up period was not extremely long, but according to the studies of W.C Gao^21^ and B. J. Wilkins and J. S. Lewis Jr.,^13^ the highest rate of recurrence occurs within the first 2–5 years from the initial treatment.

## AUTHOR CONTRIBUTIONS

**Federica Vultaggio:** Data curation; writing – original draft. **Barbara Martino:** Data curation; writing – original draft; writing – review and editing. **Letizia Nitro:** Supervision; visualization; writing – review and editing. **Emanuela Fuccillo:** Supervision; visualization. **Giovanni Felisati:** Supervision; writing – review and editing. **Loredana De Pasquale:** Data curation; funding acquisition; investigation; resources; supervision; validation.

## FUNDING INFORMATION

The authors received no specific funding for this work and they have no financial disclosures to make.

## CONFLICT OF INTEREST STATEMENT

The Authors declare that there is no conflict of interest.

## CONSENT

Written informed consent or publishing this report was obtained from the patient in accordance with the journal's patient consent policy.

## Data Availability

Data sharing is not applicable to this article as no datasets were generated or analyzed during the current study.
